# Correlation between Clinicopathology and Expression of Heat Shock Protein 72 and Glycoprotein 96 in Human Esophageal Squamous Cell Carcinoma

**DOI:** 10.1155/2010/212537

**Published:** 2010-03-10

**Authors:** Xiaoping Wang, Qiaoxia Wang, Huanping Lin

**Affiliations:** ^1^Department of Pathology, Shaanxi University of Chinese Medicine, Xianyang, Shaanxi 712046, China; ^2^Department of Infectious Disease, Xi'an Central Hospital, Xi'an, Shaanxi 710000, China

## Abstract

Heat shock protein 72 (HSP72) and glycoprotein 96 (gp96) are highly expressed in cancer tissues. Recent studies indicate the possible roles of HSP72 and gp96 in the development and progression of gastrointestinal carcinomas but detailed information is still ambiguous. We investigated the correlation between clinicopathology and expression of HSP72 and gp96 in human esophageal squamous cell carcinoma. The expression of HSP72 and gp96 was studied in 120 human esophageal squamous cell carcinomas with or without metastasis as well as in mucous membrane adjacent to cancers by way of immunohistochemistry. HSP72 immunoreactivities were detected in 112 of 120 primary tumors (93.3%) and in 30 of 120 mucous membranes adjacent to cancers (25.0%). Gp96 detected in esophageal squamous cell carcinoma and inmucous membrane adjacent to cancer was 85.0% and 20.0%, respectively. Both HSP72 and gp96 stained in cytoplasm. HSP72 and gp96 expression in esophageal squamous cell carcinomas withmetastasis was significantly higher than those with nonmetastasis (*P* < .05). The results indicate that there exists a significant correlation between the expression of HSP72 and gp96 and the progression of esophageal squamous cell carcinomas. HSP72 and gp96 expression were significantly associated with the presence of tumor infiltration, lymph node, and remote metastasis.

## 1. Introduction

The Heat shock protein (HSP) family is a highly conserved group of cellular proteins and is upregulated under stress conditions, such as heat, hypoxia, serum deprivation, neoplasia and virus infection [1–3]. It functions as molecular chaperone and biochemical regulator to mediate cell growth, apoptosis, protein homeostasis, and cellular targets of peptides [2]. Aside from their response to heat shock and chemical orphysical stress stimuli, HSPs have been reported to be overexpressed in a wide range of human tumors including breast, endometrial, ovarian, colon, lung, and prostate [4].Studies have also shown that HSP expressions have a close relationship with carcinoma prognosis [4, 5]. They may combine with oncogene products to form complexes and transport them into intracellular special sites and promote cancer cell proliferation and heterogeneous differentiation [6, 7]. Recent studies have also shown that HSP72 and gp96 are highly expressed in cancer tissues and have been used as prognostic markers in some tumors [8–12]. Study indicates the possible roles of HSP72 and gp96 in the development and progression of gastrointestinal carcinomas but detailed information is still ambiguous [13]. Esophageal squamous cell carcinoma is one of the most malignant cancers; until now there are few sensitive biomarkers in the diagnosis and prognosis of esophageal squamous cell carcinoma. There may be a correlation between the progression of esophageal squamous cell carcinoma and overexpression of HSP72 and gp96, but now there are few reports about expression of HSP72 and gp96 in esophageal squamous cell carcinoma. The present study aimed to estimate the extent of the immunohistochemical expression of HSP72 and gp96 proteins in tumoral specimens obtained from esophageal cancer patients. We also aimed to evaluate the association between the extent of expression of HSP staining and various clinicopathological parameters, tumor proliferative capacity. The results showed that there exists a significant correlation between the expression of HSP72 and gp96 and the progression in esophageal squamous cell carcinoma.

## 2. Materials and Methods

### 2.1. Immunochemistry Reagents

Mouse antihuman HSP72 monoclonal antibody was obtained from StressGen Biotechnologies (Victoria, British Columbia, Canada) and mouse antihuman gp96 monoclonal antibody was purchased from Santa Cruz Biotechnology, Inc (Santa Cruz, CA, USA). EnVisionTM kits were purchased from Dako Corp (Carpinteria, CA, USA).

### 2.2. Tissue Samples

This investigation was approved by the Ethics Committee on Human Study at Shaanxi University of Chinese Medicine (2004-4B). Paraffin specimens of primary esophageal squamous cell carcinoma from 120 patients undergoing esophageal resection were collected from the affiliated Hospital, Shaanxi University of Chinese Medicine, Xianyang, China from 2003 to 2008. None of the patients received any kind of anticancer treatment or other therapies prior to surgery. The patients consisted of 93 males and 27 females, with a mean age of 57.5 ± 6.6 years, ranging from 35 to 78 years. Among these patients, preoperative sera obtained before the start of initial treatment. The sera samples were assayed for CYFRA 21-1 SCC (squamous cell carcinoma antigen) and CEA (carcinoembryonic antigen) to evaluate the relation between the increase of tumor markers and tumor extension. Routine pathological diagnosis showed that all cases were squamous cell carcinoma. Tumors were categorized as infiltrative type in 36 (30%), massive type in 36 (26.7%), ulcerative type in 28 (23.3%) and constricting type in 24 (20%) out of 120 cases. Sixty-seven cases were well-differentiated type (grade I) and 29 cases were moderately differentiated type (grade II), while 24 cases were poorly differentiated (grade III). Among the cases, 98 cases had regional lymph node metastases, and 56 cases had remote metastases. Tumors staging was assessed using the 5th edition of the Tumor, Node, Metastasis (TNM) system according to the Union Internationale Contra la Cancrum (UICC) and the American Joint Committee on Cancer (AJCC) [14]; they were classified as T1 (*n* = 20, 16.7%), T2 (*n* = 22, 18.3%), T3 (*n* = 40, 33.3%), and T4 (*n* = 38, 31.7%). Twenty-two (18.3%) patients were node negative (N0) and 98 (81.7%) node positive (N1, *n* = 45, 37.5% and N2, *n* = 53, 44.2%). Organ metastasis was noted in 56 (46.7%) out of 120 patients examined. The specimens were fixed in 10% buffered formalin and embedded in paraffin. Serial sections, 5 *μ*m thick, were cut and placed on silane-coated glass slides.

### 2.3. Staining Methods

All sections were deparaffinized and rehydrated with graded alcohols. Endogenous peroxidase was then blocked with 3 mL/L H_2_O_2_ diluted in methanol for 30 minutes at room temperature. Antigen retrieval was performed by treating the slides in citrate buffer in a microwave for 10 minutes. The slides were incubated in a moist chamber with HSP72 mouse monoclonal antibody (1 : 100) and gp96 mouse monoclonal antibody (1 : 100) at 4°C overnight. After a complete wash in phosphate buffered saline (PBS), the slides were incubated with horseradish peroxidase labelled goat antimouse antibody (1 : 100) for 45 minutes at 37°C. After a complete wash in PBS, the slides were developed in 0.5 g/L freshly prepared 3,3*'*-diaminobenzedine solution (DAB, Sigma Co, St.Louis, Mo, USA) for 8 minutes, and then counterstained with hematoxylin, dehydrated, air dried, and mounted. Normal mouse IgG was used to substitute for the primary antibody as a negative control. No specific immunoreactivity was detected in these tissue sections. Two of the authors initially determined the fields simultaneously using a double-headed light microscope. The evaluation of HSP72 and gp96 positive cells was performed on high-power fields (×400) using a standard light microscope. Only distinctive intranuclear or intracytoplasm immunoreactivity was considered positive. In each case, more than 1000 cells were counted and the percentage of immunoreactivity was independently determined. When interobserver differences were greater than 5%, the immunostained slides were re-examined simultaneously using a using a double-headed light microscope and the percentage of positive cells were determined. When interobserver differences were less than 5%, the mean value was obtained as the positive rate. When more than 10% positive cells were detected, the case was considered positive.

### 2.4. Statistical Analysis

HSP72 and gp96 expressions differences between esophageal squamous cell carcinomas and mucous membrane adjacent to cancer were analyzed statistically using u test. The relationship between expression of HSP72 and gp96 in esophageal squamous cell carcinoma tissue with or without metastasis was analyzed statistically using *χ* ^2^ test. *P* < .05 was considered statistically significant.

## 3. Results

### 3.1. Expression of HSP72 and gp96 in Esophageal Squamous Cell Carcinomas and Mucous Membrane Adjacent to Cancer

**Figure 1:**
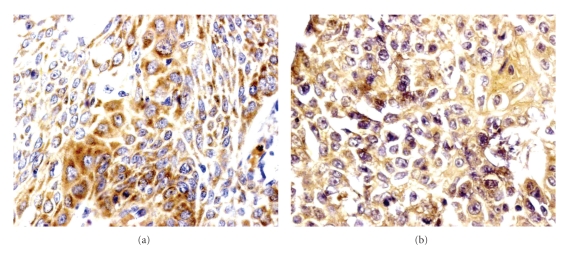
Immunohistochemistry for HSP72 and gp96 in esophageal squamous cell carcinoma cells (counterstained with hematoxylin), ×400. Distinctive intracytoplasm immunoreactivity was detected for HSP72 (a) and gp96 (b) in esophageal squamous cell cancer cells. (a): HSP72, heat shock protein 72; (b): gp96, glycoprotein 96.

The results of immunohistochemistry of HSP72 and gp96 were summarized in Table 1. HSP72 immunoreactivities were detected in 112 of 120 primary tumors (93.3%) and in 30 of 120 mucous membranes adjacent to cancers (25.0%). Gp96 detected in esophageal squamous cell carcinoma and in mucous membrane adjacent to cancer was 85.0% and 20.0%, respectively. HSP72 and gp96 proteins were mainly presenting a cytoplasmic and occasionally membraneous pattern of staining. Representative immunostainings for HSP72 and gp96 are illustrated in Figure 1. HSP72 and gp96 positive rates in esophageal squamous cell carcinoma groups were significantly higher than those in mucous membrane adjacent to cancer (*P* < .01). While in the patients prior to initial treatment, the mean concentrations of CYFRA 21-1, SCC, and CEA were 3.4 ± 2.5 (<2.0) ng/mL, 1.3 ± 1.5 (<1.5) ng/mL, and 1.5 ± 0.8 (<2.5) ng/mL, respectively. CYFRA 21-1, SCC, and CEA values were elevated in 55 of 120 (45.8%), 29 of 120 (24.2%), and 14 of 120 (11.7%) cases, respectively (Table 3).

### 3.2. Relationship between Clinicopathology and Expression of HSP72, gp96 in Esophageal Squamous Cell Carcinomas

Results showed that HSP72 and gp96 expressed higher in low differentiation of esophageal squamous cell carcinomas than that in tissues adjacent to cancers (*P* < .01). HSP72 and gp96 positive rates in lymph node metastasis and remote metastasis groups were 100%. There were significant differences of HSP72 and gp96 expressions between metastasis groups and non-metastasis groups (*P* < .05). To assess if HSPs bear a pronounced prognostic effect in patient subgroups, we conducted an extensive Kaplan-Meier analysis of HSP72 and gp96 protein expressions (low versus high). We stratified by pT stage (small versus large tumor size, pT1/T2 versus pT3/T4), tumor grading (poorly versus moderately and well differentiated), nodal status (absence versus presence of lymph node metastases, pN0 versus pN1/N2), presence of organ metastases (pM0 versus pM1), and histopathological type (intestinal versus diffuse). In cross-tables, HSP72 and gp96 expressions were significantly associated with tumor size, the presence of organ metastases and lymph node positivity (Table 2). These results suggest that there exists a significant correlation between expression of both HSP72 and gp96 and progression of esophageal squamous cell carcinomas. Alternatively, the correlations between the pretreatment serum of CYFRA 21-1, SCC, CEA and the tumor differentiation were shown in Table 4. There was no correlation between the tumor differentiation and the elevated tumor markers.

## 4. Discussion

In this study we examined the expressions of HSP72 and gp96 in 120 esophageal squamous cell carcinoma samples by immunohistochemistry. The results showed that almost all of the detected esophageal squamous cell carcinomas expressed HSP72, and majority of tumors expressed gp96, which had significant differences compared with that in mucous membrane adjacent to cancers. By way of immunohistochemistry, we found that there was a definite correlation between expression of both HSP72 and gp96 and development of esophageal squamous cell carcinomas.

The Heat shock protein (HSP) family is group of highly conserved proteins synthesized after heat induction or other stressors [1–3]. In mammalian cells, this system is divided into two predominant categories, which appear to be structurally and functionally related: the heat shock proteins (HSPs) and the glucose-regulated proteins (grps) [1]. During the growth and development of normal cells, HSP70 is constitutively expressed at low levels but the expression was dramatically enhanced by stressful conditions [2]. Heat shock protein 72, belonging to the family of HSP70, is a highly conserved protein synthesized under various stresses. In non-transformed cells at normal conditions, Hsp72 is expressed at very low levels. It is, however, present at elevated levels in the major fraction of tumors and in many transformed cell lines [15–17]. It is commonly assumed that in tumor cells the expression of HSP72 at elevated levels is the consequence of oncogenic transformation and enhanced expression of HSP72 has a close relationship with epithelial carcinoma cells growth [15–17]. Upregulated expression of the HSP70 family in tumor cells may be a requirement to serve as molecular chaperones in regulating and stabilizing oncofetal protein and mutant oncogene products during tumor growth process [18–20]. In normal cells, gp96 expressions could also be induced by various stresses to function as molecular chaperones [19, 20]. Some researchers have implied that enhanced expression of gp96 has a close relationship with cancer cells growth [11, 12]. High-level expression of gp96 could contribute to tumorigenicity of certain tumors [12, 21]. Recent studies have shown that HSP72 and gp96 are highly expressed in cancer tissues and have been used as prognostic markers in some tumors, such as hepatocellular carcinoma, gastric cancers, colonic tumors, breast cancers, and lung cancers, which have also been verified to be associated with the development and progression of the above-mentioned carcinomas [8–12]. However, few reports have studied the expression of gp96 in esophageal squamous cell carcinomas, especially during the course of tumor growth and differentiation, in simultaneous comparison with HSP72. In this experiment, we found that HSP72 and gp96 were highly expressed when esophageal squamous cell carcinomas progressed, but their roles in esophageal squamous cell carcinoma are not clear. It is reasonable to propose that HSP72 and gp96 upregulation in these tumor cells are closely related with tumor cell survival and proliferation. Recent studies have suggested that HSPs take part in cell growth and proliferation in several ways such as signal transduction and cell cycle regulation through combining certain proto-oncogene products. This indicates that these proliferating cells need higher level of HSPs to maintain the stability of tumor proteome [22, 23]. It is believed that tumor cells are a group of highly proliferative heterogeneous cells which progress gradually through mutant oncogene products [24, 25]. Continuous expression of HSP in tumor cells may be required to serve as molecular chaperones in regulating and stabilizing these products during tumor growth process. At the same time, the existence of mutant or oncogene products may stimulate HSP synthesis [6, 7].

The present study further supports the clinical significance of HSP72 and gp96 protein expression in the progression of esophageal squamous cell carcinoma. In the study, HSP72 and gp96 expressions were found to be associated with important clinicopathological characteristics for patients' management. Consistently, HSP72 and gp96 expressions were significantly associated with the presence of tumor size, lymph node and organ metastasis. In our previous studies, we have found overexpression of the HSP70, HSP72, grp94 or gp96 in human gastrointestinal carcinomas, which have some relationship with progression, invasion, and metastasis of the cancers [11, 12, 26–28]. Other researchers' studies [29, 30] also showed that overexpression of HSP70 was related to tumor configuration, lymph node metastasis, and lymphatic vessel invasion in human esophageal squamous cell carcinoma. The studies found that histopathological differentiation was significantly correlated with the expression of HSP70, while there found no significant association between HSP70 expression and patient survival. Until now sensitive tumor markers used for diagnosis of patients with esophageal squamous cell carcinoma include CYFRA 21-1, SCC (squamous cell carcinoma antigen), and CEA (carcinoembryonic antigen) [31, 32]. likely, we analyzed the patients' serum prior to initial treatment, the results indicated that the diagnostic sensitivity of CYFRA 21-1, SCC and CEA were only 45.8%, 24.2%, and 11.7%, and there were no significant correlation between CYFRA 21-1, SCC, CEA and the differentiation of esophageal squamous cell carcinomas, while the expressions of HSP72 and gp96 in esophagheal carcinoma were 93.9% and 85.0%, respectively. Our results showed that not only the expression of both HSP72 and gp96 in esophageal squamous cell carcinoma was higher than that in tissues adjacent to cancer, but also the expression of both HSP72 and gp96 in esophageal squamous cell carcinomas with metastasis was definitely higher than that of esophageal squamous cell carcinomas without metastasis. The expression of both HSP72 and gp96 in esophageal squamous cell carcinoma was related to the differentiated tissue type of esophageal squamous cell cancer. It is presumed that the combined measurement of HSP72 and gp96 in esophageal squamous cell carcinoma might be more sensitive than that of HSP70 family alone. The results indicate that upregulation of HSP72 and gp96 is likely to have some relationship with progression, invasion and metastasis of esophageal squamous cell carcinomas.

Studies revealed that considerable expression of HSPs was found in tumor cells, showing that HSPs may be induced by other stresses and participate in broader array of defenses during cell growth and cell differentiation of tumors [5–7]. Thus, it may be presumed that under various stimuli and stressful conditions, in order to avoid the damage caused by deleterious factors such as nicrosamines-oncogenesis evocator, esophageal squamous cell has to transcribe and translate high levels of HSPs in order to sustain normal metabolism and functions of cells. Under these conditions, esophageal squamous cell should synthesize HSPs rapidly to exert a protective role for esophageal squamous cells. The progression of esophageal squamous cell carcinoma is a gradual process under the long-term influence of various stimuli. During the process, inducible HSP synthesis increases gradually [33]. This viewpoint was confirmed by our results, in that HSP72 and gp96 were expressed at a higher level in esophageal squamous cell carcinoma than that in esophageal tissues adjacent to cancer. The expression levels of HSP72 and gp96 may prove useful as diagnostic or prognostic markers for esophageal squamous cell carcinoma.

Numerous investigations have been verified that HSP70 and gp96 are potent stimulators of immune responses [34–36]. The classical mechanisms of HSP70 and gp96 against tumors are believed that they may act as chaperones to facilitate major histocompatibility complex-1 (MHC-I) peptide loading, therefore increasing the tumor peptides presented by MHC-I [37–39]. Studies have shown that HSP72-associated peptides can also anchor antigen on the cell membrane and directly present it to natural killer cells or *γ* *δ* T cells as superantigen without being dependent on the stimulation of MHC-I molecules [40, 41]. Through this way cytotoxic T lymphocyte (CTL) responses could be induced and the antitumor immunity was activated. Our data shows high-level expression of HSP72 and gp96 in esophageal squamous cell carcinomas, and there was a significant correlation between their expression and progression, metastasis of tumors. These results raise the possibility that expressions of HSP72 and gp96 in esophageal squamous cell carcinomas may provide a useful link between immunity and tumor therapy against these cancers.

In conclusion, there is a close correlation between the overexpression of HSP72 and gp96 and progression of esophageal squamous cell carcinomas. The high-level expression of HSP72 and gp96 may be useful as diagnostic or prognostic markers for esophageal squamous cell carcinoma.

## Figures and Tables

**Table 1:** Relationship between immunoreactivity of HSP72 and gp96 and clinical features of Patients with esophageal squamous cell carcinomas.

Variables	*n*	HSP72 (%)	gp96 (%)	
Age (years)				*P* < .05
<60	58	52 (43.3)	48 (40)	
>60	62	60 (50)	54 (45)	

Gender				*P* < .05
Male	93	90 (75)	87 (72.5)	
Female	27	22 (18.3)	15 (12.5)	

Tumor type				*P* < .05
Infiltrative	36	34 (28.3)	32 (26.7)	
Massive	32	31 (25.8)	28 (23.3)	
Ulcerative	28	25 (20.8)	22 (18.3)	
Constricting	24	22 (18.3)	20 (16.7)	

Tumor size				*P* < .05
<5 cm	42	36 (30)	32 (26.7)	
>5 cm	78	76 (63.3)	70 (58.3)	

Lymph node metastasis				*P* < .05
Presence	98	98 (81.7)	98 (81.7)	
Absence	22	14 (11.7)	4 (3.3)	

Remote metastasis				*P* < .05
Presence	56	56 (46.7)	56 (46.7)	
Absence	64	56 (46.7)	46 (38.3)	

pT classification				*P* < .05
T1	20	16 (13.3)	14 (11.7)	
T2	22	20 (16.7)	17 (14.2)	
T3	40	38 (31.7)	35 (29.2)	
T4	38	38 (31.7)	36 (30)	

pN classification				*P* < .05
N0	22	14 (11.7)	4 (3.3)	
N1	45	45 (37.5)	45 (37.5)	
N2	53	53 (44.2)	53 (44.2)	

pM classification				*P* < .05
M0	64	56 (46.7)	46 (38.3)	
M1	56	56 (46.7)	56 (46.7)	

**Table 2:** Relationship between clinicopathology and immunoreactivity of HSP72 and gp96 in esophageal squamous cell carcinomas.

Pathologic types		HSP72		gp96	
	*n*	− (%)	+ (%)	− (%)	+ (%)
Tissues adjacent	120	90 (75.0)	30 (25.0)	96 (80.0)	24 (20.0)
to cancers					
Esophageal carcinomas^a^	120	8 (6.7)	112 (93.3)	18 (15.0)	102 (85.0)
Tumor Differentiation^b^					
Well differentiated	67	6 (9.0)	61 (91.0)	11 (16.4)	56 (83.6)
Moderately differentiated	29	2 (6.9)	27 (93.1)	4 (13.8)	25 (86.2)
Poorly differentiated	24	0 (0)	24 (100)	3 (12.5)	21 (87.5)
Lymph node metastasis^c^					
yes	98	0 (0)	98 (100)	0 (0)	98 (100)
no	22	8 (36.4)	14 (63.6)	18 (81.8)	4 (18.2)
Remote metastasis^d^					
yes	56	0 (0)	56 (100)	0 (0)	56 (100)
no	64	8 (12.5)	56 (87.5)	18 (28.1)	46 (71.9)
pT classification^e^					
T1	20	4 (20)	16 (80)	6 (30)	14 (70)
T2	22	2 (9.1)	20 (90.9)	3 (13.6)	17 (77.3)
T3	40	2 (5)	38 (95)	5 (12.5)	35 (87.5)
T4	38	2 (5.26)	38 (100)	2 (5.3)	36 (94.7)
pN classification^f^					
N0	22	8 (36.4)	14 (63.6)	18 (81.8)	4 (18.2)
N1	45	0 (0)	45 (100)	0 (0)	45 (100)
N2	53	0 (0)	53 (100)	0 (0)	53 (100)
pM classification^h^					
M0	64	8 (12.5)	56 (87.5)	18 (28.1)	46 (71.9)
M1	56	0 (0)	56 (100)	0 (0)	56 (100)

^a^ *P* < .01, ^b^ *P* < .01, versus tissues adjacent to cancers; ^c^ *P* < .05, ^d^ *P* < .05, versus non-metastasis groups; ^e^ *P* < .05, pT1,T2 versus pT3,T4; ^f^ *P* < .05, pN1,N2 versus pN0; ^h^ *P* < .05, pM1 versus pM0.

**Table 3:** Rates of positivity for CYFRA 21-1, SCC, and CEA in pretreatment serum samples from patients with esophageal squamous cell carcinoma.

Marker	Number	Mean ± SD	Cutoff	Sensitivity
		(ng/ml)	(ng/ml)	(%)
CYFRA 21-1	120	3.4 ± 2.5	<2.0	55 (45.8)^a^
SCC	120	1.3 ± 1.5	<1.5	29 (24.2)
CEA	120	1.5 ± 0.8	<2.5	14 (11.7)

^a^ *P* < .05, versus CEA.

**Table 4:** Tumor markers in pretreatment sera samples and tumor differentiation of the patients.

		Tumor differentiation (%)			
	*n*	Well differentiated	Moderately differentiated	Poorly differentiated	
CYFRA21-1	(−) 65	14 (21.5)	20 (30.8)	31 (47.7)	
	(+) 55	12 (21.8)	17 (30.9)	26 (47.3)^a^	*P* > .05

SCC	(−) 91	20 (22.0)	33 (36.3)	38 (41.7)	
	(+) 29	7 (24.1)	10 (34.5)	12 (41.4)^b^	*P* > .05

CEA	(−) 106	28 (26.4)	37 (34.9)	41 (38.7)	
	(+) 14	4 (28.6)	5 (35.7)	5 (35.7)^c^	*P* > .05

^a^ *P* > .05, versus CYFRA21-1 negative group; ^b^ *P* > .05, versus SCC negative group; ^c^ *P* > .05, versus CEA negative group.
